# Jan Evangelista Purkynje (1787–1869)

**DOI:** 10.1007/s00415-013-7184-8

**Published:** 2013-11-16

**Authors:** Andrzej Grzybowski, Krzysztof Pietrzak

**Affiliations:** 1 Department of Ophthalmology, Poznań City Hospital, ul. Szwajcarska 3, 61-285 Poznan, Poland; 2 Department of Ophthalmology, University of Warmia and Mazury, Olsztyn, Poland; 3 Department of Orthopaedics and Traumatology, University of Medical Sciences, Poznan, Poland

**Fig. 1:**
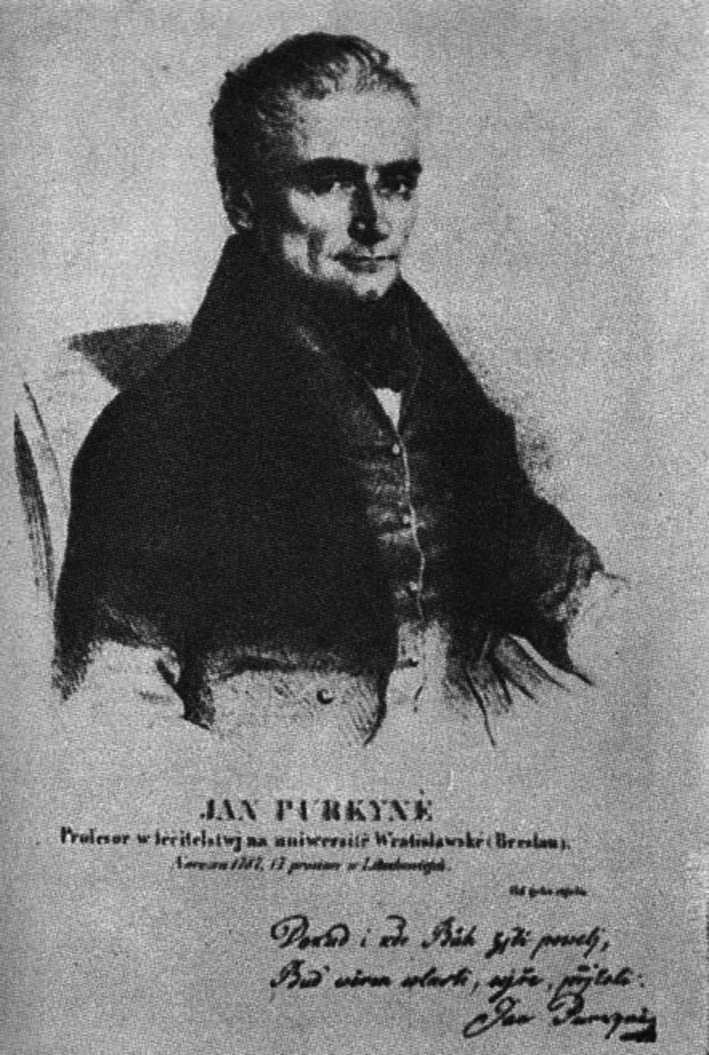
Jan Evangelista Purkynje (1787–1869)

Jan Evangelista Purkynje (Fig. 1) was born on December 17, 1787, in Libochovice, in what was then the Czech territory in the Austro-Hungarian monarchy. His father was an estate manager. After his father’s death when Jan was 6 years old, he was encouraged to become a priest. These plans along with his own poverty led to a situation in which, from the age of 10, he was driven from one Piarist monastery school to another, learning German and Latin along the way. He was sent to the Piarist Philosophical Institute in Litomysl, and later, the Philosophical Institute in Prague. As a fresh graduate of Prague’s Institute, he had to earn money as a tutor of rich children. In 1813, he took up medical studies at the University of Prague. In 1818, he graduated from the medical faculty. He obtained a doctorate in 1819, following a thesis on subjective visual phenomena [1]. By way of self-examination, he established that the visual sensations are caused by brain activity and the brain's connection to the eye, such that they might not be triggered by external stimulation. He became a prosector and an assistant in the Physiology Institute at the University of Prague, but he had no opportunities to carry out his own experiments. He conducted research on vertigo phenomena, still relying on the method of self-examination, in a Prague fairground on a carousel. He noticed that the vertigo direction is independent of the direction of rotation, but depends instead on the position of the head in relation to the body. Additionally, he described the phenomena of nystagmus [2]. He also analyzed the physiological phenomena that occurred after the use of certain drugs, including camphor, opium, digitalis and belladonna. He experimented on himself, sometimes going to dangerous extremes. He noticed that using one drug after another seemed to intensify the effect of the first one. He observed, nearly 30 years before Helmholtz, the interior of the eye in the light reflected into it by concave lenses. He noticed some differences of color detection in dim light, especially in comparison with the detection in daylight [3]—what was then called the “Purkynje phenomenon”. Nowadays, it is explained by differential rod and cone excitation. He also emphasized the significance of fingerprints in crime detection, an idea that was an absolute innovation at that time.

Purkynje applied for a teaching position at many universities in the Austrian Empire. However, he was unsuccessful on many occasions. He was a Czech, and university officials preferred to promote German citizens to academic positions. Fortunately, his doctorate thesis was well received, and caught the attention of Goethe, who was interested in the same issue. With strong support shown by Goethe and Aleksander von Humboldt, in 1823, he was offered the position of the Professor of Physiology at the University of Breslau. His candidacy was accepted despite strong opposition from the faculty members. Thus, the most fruitful period of his career began.

Purkynje’s successes in Breslau were based on excellent equipment and new techniques for the preparation of research material. He had a very modern and accurate microscope and microtome. He was the first to establish that the whole body is composed of cells [4]. He did this 2 years ahead of T. Schwann. Paradoxically, in the history of science, Schwann is more commonly connected with this discovery. This may have resulted from the fact that Purkynje's main interest was the inside of the cell, while Schwann described the cell membrane and was the first to use the word “cell”. Undoubtedly, Purkynje was the first to observe and account for the cell nucleus [4]. He also noticed that cells are the structural components of animals and plants. He introduced the terms “protoplasm” of the cells, and “plasma” of the blood into the scientific language.

The modern techniques of Purkynje's time allowed him to obtain his neurological results. In 1837, he published a paper about the ganglion cells in the brain, spinal cord, and cerebellum [5]. He was the first to notice the significance of the grey substance of the brain. Before his discovery, scientists thought that only the white substance and nerves had any meaning. He emphasized that those cells are the centers of neurological function and that nerve fibers are like wires that transmit power from the nerves to the whole body. He accurately described the cells in the middle layer of the cerebellum with dendrites branching like a tree. They were then called Purkynje cells [5, 6]. Purkynje’s discoveries were often published in the dissertations of his assistants. He supervised the doctorate of David Rosenthal (1821–1875); they jointly discovered that nerves have fibers inside [7], and analyzed the number of nerve fibers in spinal and cranial nerves [7]. Purkynje also established that sleep is caused by a decrease of external impulses [8]. He conducted research on the effects of partial destruction of the animal brain by needles, being one of the earliest researchers to use this method. For many years, Purkynje used a special rotating chair and recorded all the optical, motion-associated, and physiological signs accompanying vertigo. He carried out studies in which he directed the galvanic current flow through his own skull, and observed the resulting vertigo and physiological phenomena.

He determined the movement of cilia in the genital and respiratory systems, and ultimately, in the ventricles of the brain as well [9]. In 1839, Purkynje discovered the fibrous tissue that transmits electrical impulses from the atrioventricular node to the ventricles of the heart [10]. Today, they are called the Purkynje fibers.

In 1839, Purkynje opened the Physiological Institute in Wrocław, which was the first such institute in the world. He became the dean of the medical faculty, elected to this position four times in a row. In 1850, he became a professor of physiology at the University of Prague. There, he concentrated on encouraging a return to the use of the Czech language instead of German in the university's operations.

In 1827, Purkynje got married to Julie Rudolphi, the daughter of a professor of physiology from Berlin. They had four children, two of whom were girls that died in early childhood. After 7 years of marriage, Julie died, leaving Purkynje with two young sons and in deep despair. Purkynje died on July 28, 1869, in Prague. He was buried in the cemetery for distinguished citizens near the Czech Royal Castle on Wyszehrad.
